# Hallucinations and conscious access to visual inputs in Parkinson’s disease

**DOI:** 10.1038/srep36284

**Published:** 2016-11-14

**Authors:** Stéphanie Lefebvre, Guillaume Baille, Renaud Jardri, Lucie Plomhause, Sébastien Szaffarczyk, Luc Defebvre, Pierre Thomas, Christine Delmaire, Delphine Pins, Kathy Dujardin

**Affiliations:** 1 Univ. Lille, CNRS, UMR 9193 - SCALab - Sciences Cognitives et Sciences Affectives, F-59000 Lille, France; 2 CHU Lille, Clinique de Psychiatrie, CURE, F-59000 Lille, France; 3 Department of Neurology and Movement Disorders, Lille University Medical Center, F-59000 Lille, France; 4 Univ. Lille, Inserm, U1171 - Degenerative & vascular cognitive disorders, F-59000 Lille, France; 5 Neuroimaging Department, Lille University Medical Center, F-59000 Lille, France

## Abstract

The pathophysiology of visual hallucinations in Parkinson’s disease has yet to be characterized. Although stimulus-driven (“bottom-up”) processes are known to be impaired, the role of “top-down” processes remains to be determined. Distinguishing between conscious and non-conscious detections (i.e. access to consciousness) may be a valuable way of monitoring top-down processes. Conscious access to visual inputs was investigated to identify the neural substrates underlying susceptibility to hallucinations in Parkinson’s disease. Seventeen healthy controls, 18 Parkinson’s disease patients with minor visual hallucinations and 16 without were enrolled in the study. During functional magnetic resonance imaging, the participants performed a visual detection task. The detection threshold was significantly higher in each patient group than in healthy controls while the two groups of patients did not differ significantly. Compared with hallucination-free patients, patients with minor hallucinations displayed hyperactivation of prefrontal and right occipital cortices, and hypoactivation of the left cingulate, temporal and occipital cortices. During conscious access to visual inputs, the functional network in patients with visual hallucinations differed from that seen in patients without visual hallucinations. This suggests that the supremacy of top-down processes in visual information processing may enhance susceptibility to hallucinations in Parkinson’s disease.

Over the course of Parkinson’s disease (PD), visual hallucinations (VHs) are frequently reported (17 to 72%)^1^. VHs may be minor (i.e. misperception of an existing object or confusion between people) or complex (e.g. elaborate visual scenes)^2^. The severity of the VHs depends not only on their frequency but also on the extent to which the patient believes in the hallucinations. This neuropsychiatric disorder has a marked socio-economic impact, since it accounts for the leading cause of institutionalization in PD patients and is associated with a higher mortality rate^3^,^4^. Furthermore, the risk of developing dementia is four times higher in PD patients with VHs than in those without^5^. Given that the pathophysiology of VHs in PD remains unclear, treatment is difficult and poorly effective.

Recently, an MRI study reported atrophy of the temporo-occipital regions in PD patients with VHs (independently of their cognitive status)^6^. These areas are involved in visual recognition based on the saliency of a stimulus (a “bottom-up” process). In view of the reduced visual input in PD patients (due to both a lack of dopaminergic cells in the retina and neurodegeneration in temporo-occipital areas), Diederich *et al*.^2^ proposed a model of VHs in which memory information associated with “waking dreams” is “unblocked” in order to compensate for the lack of visual information. This could be the starting point for visual phenomena. This integrated model is supported by post-mortem evidence of abnormally dense deposits of alpha-synuclein in the temporal gyri and transenthorinal cortex of PD patients with VHs^7^,^8^. In addition to bottom-up processes, the processing of visual information is also based on top-down processes that take account of context and the subject’s prior knowledge and are related to voluntary attention and mental representations of a stimulus. Top-down processes involve the pre-frontal and parietal areas of the cortex. Recently, a functional MRI (fMRI) study of a patient with PD reported hyperactivation of the frontal areas, thalamus and midbrain and hypoactivation of temporo-occipital regions during complex VHs^9^. Furthermore, several studies have suggested that impairments in reality monitoring have a role in the occurrence of VHs^10^,^11^. A failure of the networks regulating attention was also suggested^12^,^13^. According to Dehaene *et al*.^14^, these top-down processes have a key role in “access to consciousness” (distinction between conscious and non-conscious detection of a stimulus). A number of pioneering studies have defined the brain network associated with conscious access to visual inputs in healthy subjects. This network involves brain areas (including the fusiform, lateral occipital, prefrontal and parietal cortices) that are strongly correlated with visual conscious perception, whereas the primary visual cortex shows constant brain activation with both conscious and unconscious visual percepts^15^,^16^,^17^. Moreover, MRI studies in PD patients with VHs have revealed both structural and functional abnormalities in prefrontal regions linked to conscious access to visual inputs^18^,^19^. However, the role of access to consciousness of visual information in the occurrence of hallucinations in PD has not previously been investigated. We hypothesized that modification of this process by frontostriatal and cortical dysfunction^20^ might tonically change visual information processing in PD patients and thus favor the occurrence of hallucinations.

The primary objective of the present study was to expand our knowledge on the mechanisms of minor VHs in PD. To this end, we studied the neural networks involved in conscious access to visual inputs during a perceptive task similar to that developed by Pins and ffytche^17^. We assumed that PD patients with minor VHs have impaired conscious access to visual inputs. This impairment might be characterized by a higher threshold for visual detection in PD patients with minor VHs than in patients without VHs or healthy subjects. Lastly, we hypothesized that the difference in the visual detection threshold is associated with differential brain activation in frontoparietal and temporo-occipital networks.

## Results

### Behavior

**Figure 1:**
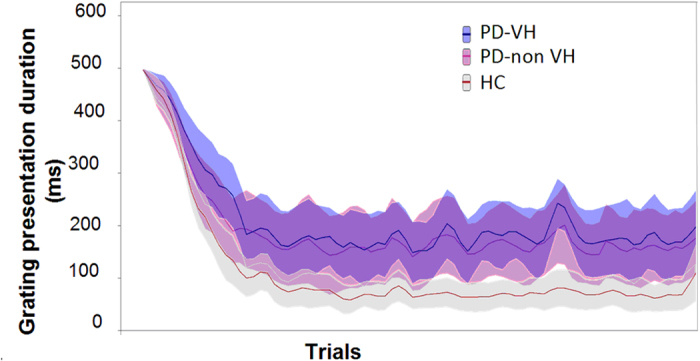
The visual detection threshold Mean ± SD grating duration for each of the 70 trials and for each of the 3 groups. PD-nonVH: PDpatients without visual hallucinations; PD-VH: PD patients with visual hallucinations; HC: healthy controls.

For the visual detection threshold (results illustrated in Fig. 1), the Kruskal-Wallis test demonstrated a statistically significant difference between the 3 groups (F = 5.072, *P* = 0.01). In fact, the post-hoc test demonstrated that the threshold was lower in the Healthy Control (HC) group (69.29 ± 55.99 ms) than in the PD patients with minor VHs (PD-VH) group (175.17 ± 124.12 ms; *P* = 0.0025) and the PD patients without VHs (PD-nonVH) group (163.38 ± 129.72 ms; *P* = 0.007). No difference in the visual detection threshold was observed when comparing the two groups of patients (*P* = 0.35).

The threshold estimation experiment produced approximately equal numbers of “seen” and “unseen” trials for the 3 groups, with respectively 21 ± 3 seen at the threshold (ST) and 20 ± 3 unseen at the threshold (UT) trials for the HC group; 20 ± 4 ST trials and 19 ± 4 UT trials for the PD-nonVH group and 18 ± 3 ST trials and 19 ± 4 UT trials for the PD-VH group. Furthermore, the participants in all 3 groups performed the task correctly. The mean error rate was less than 1 in 5 for both the positive and negative controls, with respectively 0.355 ± 1.05 errors for the 5 positive controls and 0.24 ± 0.44 errors for the 5 negative controls in the HC group, 0.25 ± 0.45 and 0.56 ± 1.03 errors in the PD-nonVH group and 0.67 ± 1.19 and 0.78 ± 1.17 errors in the PD-VH group).

No difference between the three groups was observed in terms of performance in the visuospatial function tests (Table 1). Furthermore, the visual detection threshold was not significantly correlated with the judgment of line orientation test score (r = −0.09; *P*_*B*_ (Bonferroni corrected) > 0.9) or the visuoconstruction test score (r = 0.09; *P*_*B*_ > 0.9). In the PD-VH group, the visual detection threshold was not significantly correlated with the score on the various hallucination scales (*University of Miami Parkinson’s Disease Hallucination Questionnaire,* visual modality: r = −0.185; *P*_*B*_ > 0.9; VH frequency: r = −0.35; *P*_*B*_ = 0.45; VH severity: r = −0.07; *P*_*B*_ > 0.9).

### fMRI results

#### Whole-brain ANCOVA

##### Main effects

The whole-brain ANCOVA revealed significant main effects of group, fMRI condition and the interaction between the two [at q(FDR) = 0.05: fMRI condition F (4,192) = 2.97; group: F (2,48) = 9.17; interaction F (8,192) = 5.11].

#### Post-hoc analyses

**Figure 2:**
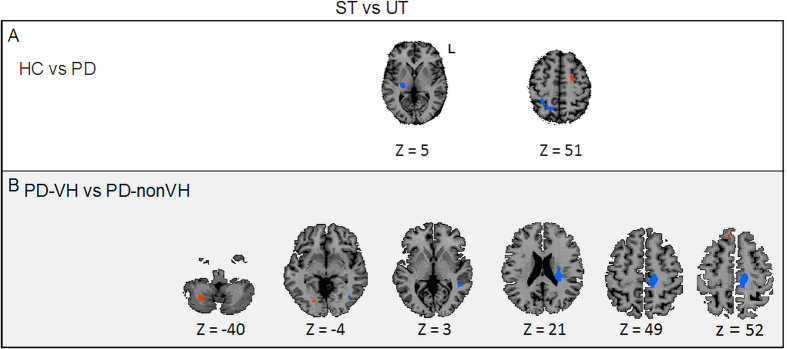
Whole-brain **ANCOVA** A whole-brain ANCOVA, comparing conscious access to visual inputs in the three groups [t (200) = 2.59, *P* = 0.01 (corrected at the cluster level)]. Panel A: Comparison of conscious access to visual inputs in the HC vs. PD groups. Panel B: Comparison of conscious access to visual inputs in the PD-VH vs. PD-nonVH groups. ST: seen trials at the threshold, UT: unseen trial at the threshold, PD-nonVH: PD patients without visual hallucinations; PD-VH: PD patients with visual hallucinations; HC: healthy controls; L: left.

*Group-comparisons for the [ST-UT] contrast*. When comparing HCs with PD patients (Fig. 2A, Table 2), three areas were differentially activated at the visual detection threshold, [t (200) = 2.59, *P* = 0.01 (corrected at the cluster level)]: the right thalamus and the right precuneus were more involved in PD patients than in HCs, and the left dorsal premotor cortex (PMd) was more involved in HCs than in PD patients.

When comparing the PD-VH and PD-nonVH groups (Fig. 2B, Table 2), six areas were differentially activated at the visual detection threshold [t (200) = 2.59, *P* = 0.01 (corrected at the cluster level)]: the right cerebellum and the right occipital cortex were more involved in the PD-VH group than in the PD-nonVH group, and the left cingulate cortex, the left caudate nucleus, the left temporal cortex and the left occipital cortex were more involved in the PD-nonVH group than in the PD-VH group. No significant correlation was observed between the observed activations and the severity, frequency and intensity of visual hallucinations.

## Discussion

The main findings of the present study were as follows. Firstly, PD patients displayed a higher visual detection threshold than HCs. This difference was associated with reorganization of the brain network involved in conscious access to visual inputs. Secondly, the brain network involved in conscious access to visual inputs in PD-VH patients differed from that involved in PD-nonVH patients - despite similar visual detection thresholds. In particular, PD-VH patients showed hyperactivation of the frontal regions.

Our behavioral results evidenced a significantly higher visual detection threshold in PD patients than in HCs. This finding suggests the presence of an impairment related specifically to PD and not attributable to age (since the patients and the HCs were matched for age). This observation is in line with the observation of lower visual contrast acuity in patients with PD than in HCs^21^. When comparing ST and UT conditions, PD patients displayed concomitant hyperactivation of the right thalamus and precuneus and hypoactivation of the left PMd (relative to HCs).

Recruitment of the precuneus during conscious access to visual inputs may be linked to the structure’s involvement in a variety of highly integrated functions, including visuospatial imagery^22^. Moreover, it has already been demonstrated that the precuneus is associated with visual awareness. For example, Kjaer *et al*.’s PET study of cerebral blood flow^23^ highlighted the differential recruitment of the right precuneus and dorsolateral prefrontal cortex when stimuli lasted long enough to elicit awareness. In PD patients, greater recruitment of the right precuneus might be required for stimuli having access to consciousness - leading to a hyperactivation of this region.

Some of the areas highlighted in the present study might be functionally linked to performance of the task *per se*, rather than involved in conscious access to visual inputs. This might have been the case for the thalamus and the PMd. Hyperactivation of the right thalamus mainly concerned the lateral part (namely the ventral lateral nucleus and the pulvinar). The ventral lateral nucleus is part of the thalamus’ motor function region^24^,^25^,^26^ and has many connections with the motor cortex. It is known that the ventral lateral nucleus has a role in movement planning and coordination. The right-side hyperactivation of this thalamic nucleus observed here is therefore difficult to explain, since it was observed in a time window during which the participants were preparing to press a response button with their right hand. The pulvinar constitutes the associative part of the thalamus. It integrates information from the other thalamic nuclei and has many connections with the associative cortex^27^. The pulvinar has a central role in visual attention and is a key component of the visual attention network^28^. The hyperactivation of the pulvinar observed in the present study might be related to the fact that the visual detection task was more demanding for PD patients than for HCs and thus required more cognitive resources.

The PMd’s involvement might be due to its well-known involvement in decision-making processes in general and the selection of an action to be executed in particular^29^,^30^. Hypoactivation of the PMd in PD patients is also well established. Indeed, this region is abnormally active when PDpatients are in the “off-drug” state, with an increase in the movement-related BOLD signal in the PMd and the parietal posterior cortex (compared with HCs)^31^. This abnormally intense PMd activity is thought to reflect changes in motor cortical excitability in PD, although chronic dopaminergic stimulation reduces the responsiveness of corticocortical circuits^32^. In the present study, all patients were assessed in the “on-drug” state and had been treated for several years (the mean disease duration was 8 and 9 years in the PD-VH and PD-nonVH groups, respectively). During the visual detection task, the participants responded with their right hand. Thus, left PMd hypoactivation might reflect less efficient response selection in the PD patients (relative to HCs).

Despite a similar visual detection threshold in the two patient groups, the brain network associated with conscious access to visual inputs by the PD-VH group differed from that in the PD-nonVH group. When comparing the ST and UT conditions, the PD-VH group showed hyperactivation of the right cerebellum and the prefrontal and right occipital cortices, and hypoactivation of the left cingulate, temporal and occipital cortices and left caudate nucleus (relative to the PD-nonVH group).

Hypermetabolism in prefrontal areas has already been observed in PD patients suffering from VHs^33^, and may reflect an attempt to compensate for cognitive impairments. Hyperactivation of frontal regions was also found in PD patients with VHs during kinematic visual stimulation with a stroboscope^34^. At the opposite, Shine *et al*. observed that PD patients with visual hallucinations failed to significantly activate the regions of the dorsal attention network (including the dorsal prefrontal cortex) when processing ambiguous visual stimuli. However, in their study, PD patients were classified as hallucinators not on clinical criteria but on the basis of their performance at a bistable perception paradigm used for the fMRI scan^35^. In the present study, hyperactivation of frontal regions was linked to conscious access to visual inputs by patients who were susceptible to VHs (i.e. in agreement with our initial hypothesis).

The hypoactivation of the left occipital and temporal cortex observed in the PD-VH group is also in line with our initial hypothesis and might reflect both low involvement of executive and attentional functions and impaired processing of visual inputs. In fact, this observation agrees with the findings of a structural MRI study in which atrophy of the left occipital cortex was observed in PD patients with VHs^18^. Hypoactivation of the temporo-occipital regions^9^ has also been observed in PD patients during the occurrence of VHs. Hyperactivation of the right occipital cortex is more difficult to explain. One possible explanation is a compensatory (but not sufficiently efficient) right-side activation in response to a left-side impairment. This hypothesis is reinforced by our observation of overall hypoactivation of the left hemisphere and (probably compensatory) right-side hyperactivation in the PD-VH group (relative to the PD-nonVH group). Access to consciousness in the PD-VH group was also characterized by hypoactivation in the cingulate and the caudate nucleus, suggesting impaired involvement of executive and attentional functions^36^,^37^,^38^,^39^.

Given that the mean severity of the motor symptoms was similar in the PD-VH and PD-nonVH groups, hyperactivation of the right cerebellum in PD patients with VHs is unlikely to be related to the motor response required by the task. There are few data on hemodynamic changes in the cerebellum in PD patients with VHs. Although the cerebellum and the basal ganglia are highly interconnected^40^, the cerebellum has long been considered as a major component of the movement control circuitry. Nevertheless, a role in human cognition has also been suggested following the observation of cerebellar activation during various cognitive tasks (for a review, see ref. 41). In terms of visual information processing, Kellermann *et al*. showed that the cerebellum is involved in the attentional modulation of bottom-up V5 afferents to the posterior parietal cortex and the dorsal visual stream^42^. Yao *et al*. measured the amplitude of low-frequency fluctuations (ALFF, a functional index related to glucose metabolism and local field potentials) during resting-state fMRI of PD patients with and without VHs and of HCs^43^. They reported that the ALFF in the temporo-parietal regions, the medial temporal gyrus and the cerebellum were greater in PD patients with VHs than in patients without VHs or HCs. Yao *et al*. considered that this enhanced cerebellar hemodynamic response may reflect an attempt to compensate for higher-order cortical impairments. In the present study, the greater right cerebellum activation associated with access to consciousness in PD patients with VHs might also reflect a means of compensating for abnormal visual input processing.

In summary, we suggest that reorganization of this brain network especially reflects dysfunction of the frontoparietal and temporo-occipital areas associated with susceptibility to hallucinations in Parkinson’s disease.

The present study advanced our understanding of the neural substrates of susceptibility to hallucinations in PD. One of the novel aspects of our study was the inclusion of patients with minor VHs, based on a strict assessment of VH severity via a detailed questionnaire (the UMPDHQ). Most studies on the pathophysiology of VHs in PD only included patients with complex VHs, and some of the latter already displayed cognitive impairments. In the present study, none of the PD-VH patients was demented. As shown in Table 1, the participants in the PD-VH group had a good cognitive status and did not differ from the HC and PD-nonVH groups in this respect. Hence, when seeking to explain the observed differences in conscious access to visual inputs, we were able to rule out cognitive decline as a confounding factor. Moreover, we were able to include a substantially larger group of PD patients (18 in the PD-VH group and 16 in the PD-nonVH group) than previous studies in the field. Strict matching with HCs for age and education duration further strengthened our findings.

Our study was the first to use an event-related visual detection task to investigate functional correlates of access to consciousness in PD. The task had been previously used to investigate the neural bases of conscious access to visual inputs in young, healthy adults^17^ and VH-free patients with schizophrenia (*personal communication*). The task is independent of the subject’s educational, age and motor status, and the results for a given individual are highly reproducible. Control trials enabled us to ensure that the task had been completed successfully. Furthermore, this procedure allowed us to evaluate brain activation patterns associated with conscious access to visual inputs independently of inter-individual differences in behavioral performance, since the threshold (and thus the trials at the threshold) was defined individually for each participant.

The present study had several limitations. Firstly, although we include an exceptionally large group (for an fMRI study) of well-characterized, strictly matched PD patients with and without VHs, the absolute sample size may be considered to be quite small and thus reduced the study’s statistical power. Accordingly, we were not able to use a very stringent significance threshold for our statistical analyses and thus cannot fully rule out the possible occurrence of type I errors. In fact, for all the RFX analyses, a cluster-level corrected threshold of *P* < 0.01, alpha level = 0.05 was applied. Even if this method constitutes a standard in similar clinical contexts to control for multiple comparisons, a recent paper^44^ suggests that cluster inference may lead to false positive results. Here, we nevertheless referred to this method, given the difficulties associated with the parkinsonian population that shows fatigue and additional movements inside the scanner leading to an increase in BOLD signal variability. Readers should remember that “70% chance of finding at least one false positive” does *not* mean that “70% of positives are false”. If there are lots of true positives, only a minority of positives will be false. However, it is impossible to directly know the true positives rate. Although, all these statistical considerations are very important, dealing with pathological considerations such as neurological disorders requires some flexibility both in terms of samples size or of corrections’ level to overcome the inherent difficulties in testing these populations (age, movements, …). By consequence, even if our results need further confirmation, they are both informative and relevant for the field.

Secondly, our subjects had not undergone an in-depth eye examination and vision test. Given that contrast acuity is known to be abnormally low in patients with PD^21^,^45^, we cannot rule out the possibility of bias in our data as a result (at least in part) of sensory impairments associated with PD. Nevertheless, we checked that none of the included patients had impairments of visual acuity, eye sockets and eye movements. Moreover, another study of patients with PD did not find that contrast acuity differs as a function of the presence or absence of VHs^35^.

Lastly, to avoid any interference from motor impairment, the PD patients were assessed and scanned in the “ON” drug state and we cannot exclude that dopaminergic treatments have modified metabolism repartition and so may have perturbed BOLD signal during the task.

## Conclusion

The visual detection task used in the present study enabled us to evaluate conscious access to visual inputs in Parkinson’s disease patients with VHs. Even though patients with and without VHs had similar visual detection thresholds, susceptibility to hallucinations in Parkinson’s disease was associated with a temporo-occipital impairment and over-recruitment of prefrontal areas during conscious access - suggesting that the supremacy of top-down mechanisms in visual information processing may enhance susceptibility to hallucinations in Parkinson’s disease.

## Materials and Methods

### Population

This study was approved by the local investigational review board (*CPP Nord-Ouest IV*, Lille, France; reference: 2013-A00531–44) and was performed in accordance with the provisions of the Declaration of Helsinki. After providing informed consent, 51 participants were enrolled (Table 1) and allocated to one of three subgroups: healthy controls (HCs: n = 17), PD patients with minor VHs (PD-VH: n = 18), and PD patients without VHs (PD-nonVH: n = 16).

The presence of VHs was confirmed by the hallucination item of the Neuropsychiatric Inventory-Clinician. The severity and frequency of VHs were rated on a visual analog scale, and the intensity of VHs was assessed by using the University of Miami Parkinson’s Disease Hallucinations Questionnaire. Patients with PD were allocated to the PD-VH group if they experienced minor VHs (sensations of passage/presence or misperception, less than twice a day) or to the PD-nonVH group if they had never experienced VHs.

All participants were aged between 40 and 80 and had normal or corrected-to-normal vision. The exclusion criteria were (i) contraindications to MRI, (ii) inability to perform the task or understand the instructions, (iii) dementia (according to the Movement Disorders Society criteria for PD patients^46^ and DSM-IV for healthy controls; score on the Mini Mental State Examination <26 and performance at a comprehensive neuropsychological test battery) or a psychiatric disorder, (iv) pregnancy and (v) change in psychotropic medications in the 30 days prior to inclusion. We also excluded HCs with a history of neurological or psychiatric disorders and PD patients treated with deep-brain stimulation. To deal with the issue of cognitive impairment, all the participants underwent a comprehensive neuropsychological assessment including tests representing the five main cognitive domains (details are shown in Table 1) according to the guidelines of the Movement Disorders Society taskforce on mild cognitive impairment in Parkinson’s disease^47^,^48^.

On inclusion, the patients’ medications, severity of motor and non-motor symptoms were recorded (Supplementary file 1). None of the PD patients had previously been treated with clozapine-like or other antipsychotics.

### Study design

Each participant took part in two separate sessions. In the first session, neurological and neuropsychological assessments were followed by a short period of training on the visual detection task. The second session was dedicated to MRI and was divided in three stages: (i) vision correction and selection of MRI-compatible spectacles, (ii) familiarization with the inside of the scanner, the MRI environment and the use of the MRI-compatible response pad (Cedrus LU 400-PAIR, Cedrus Corporation, San Pedro, CA 90734 - USA), and (iii) MRI data acquisition.

### Stimuli

A sinusoidal, circular grating of 3 cycles per degree (subtending a visual angle of 7.3° and with a mean luminance of 3.5 cd/m^2^) was displayed at the center of a grey screen (with the same mean luminance as the grating). To focus the participant’s attention and reduce eye movements, a grey fixation cross was displayed in the center of the screen (also the center of the grating). For training purposes (outside the MRI scanner), the stimulus was presented on a computer monitor (MacBookPro a1278, 13-inch display, refresh rate: 60 Hz; resolution: 1280 × 800 pixels; computer-eye distance: 60 cm). In the MRI scanner, the stimulus was back-projected onto a 600 × 400 mm screen (using a Toshiba TLP 450E LCD projector; resolution: 1280 × 800 pixels; refresh rate: 60 Hz, screen eye-distance: 120 cm) placed at the end of the bore (behind the participant’s head) and viewed via an angled mirror.

### Visual detection task

This method consisted in modifying the duration of stimulus presentation from trial to trial, on the basis of the participant’s previous response. At the beginning of the task, the presentation duration decreased after a “Yes” response (“I saw the grating”) and increased after a “No” response (“I did not see the grating) in 70 ms steps. This procedure enables the participant to reach the visual threshold in fewer trials. After a “No”-“Yes”-“No” or “Yes”-“No”-“Yes” sequence of responses, the steps changed to 16 ms. These smaller steps enabled the threshold to be determined more precisely. Two independent, randomly interleaved series were administered. In each series, the initial presentation duration was 500 ms. The objective of this procedure was to obtain a large number of trials at threshold and thus maximize the power of fMRI statistical tests.

**Figure 3:**
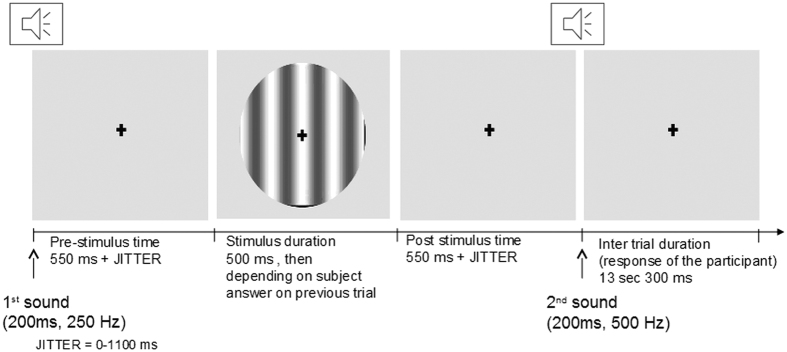
Design of the threshold evaluation study. The visual detection task consisted of 70 threshold evaluation trials. Each threshold evaluation trial began with a sound signal (duration: 200 ms; frequency: 250 Hz). The stimulus was then presented after an interval of 550 ms (the pre-stimulus interval) plus a random jitter time ranging from 0 to 1100 ms). After a second sound signal (duration: 200 ms, frequency: 500 Hz, also presented after an interval of 550 ms plus a random jitter time ranging from 0 to 1100 ms), the subject was required to press one of two buttons on the response pad with the right hand to indicate whether or not he/she had seen the stimulus. The inter-trial interval was 13300 ms.

Each trial (Fig. 3) was composed of three steps: (i) a sound (250 Hz, 200 ms) announcing the start of the trial, (ii) presentation of the stimulus after an interval of 550 ms (the pre-stimulus interval) plus a random jitter time ranging from 0 to 1100 ms (ensuring that participants could not anticipate the appearance of the stimulus), and (iii) a sound (500 Hz, 200 ms, also presented after an interval of 550 ms plus a random jitter time ranging from 0 to 1100 ms) indicating that the participant had to press one of the two buttons (“Yes” or “No”) on the response pad with the right hand to indicate whether or not they had seen the stimulus. The fixation cross was displayed throughout the visual detection task. During the training inside the MRI scanner, an inter-trial interval of 13300 ms was used (giving a trial duration of 16 seconds), whereas it was reduced to 3300 ms during the training outside the MRI scanner (giving a trial duration of 6 seconds).

The control trials enabled us to check whether or not participants were following the task instructions. The overall design was similar but in 5 of the control trials (the negative controls), nothing was presented on the screen (grating contrast: 0%). In the 5 other control trials (positive controls), a highly visible stimulus (grating contrast: 100%) was displayed.

The training outside the MRI scanner comprised 40 threshold evaluation trials and 10 control trials. The training inside the MRI scanner comprised 10 threshold evaluation trials (+2 control trials) and the entire visual detection task of 70 threshold evaluation trials (+10 control trials).

### Behavioral analysis

For each participant, the 50% visual detection threshold was determined using the following method. A linear regression analysis was performed on each set of 5 consecutive trials. Trials were considered to be at the threshold when the slope of the regression line was zero. All other trials were classified as being “not at the threshold”. Based on this classification and the participants’ responses, the trials were divided into 5 categories: (i) those seen at the threshold (ST), (ii) those unseen at the threshold (UT), (iii) those seen not at the threshold (SNT, including positive control trials), (iv) those unseen not at the threshold (UNT), and (v) other trials (OTs, including negative control trials and error trials, i.e. when participants failed to reply or made a mistake in positive control trials). The visual detection threshold was defined for each subject as the mean duration of stimulus presentation for trials at the threshold (ST and UT).

A Kruskal-Wallis test was used to compare the groups’ respective visual detection thresholds (HCs vs. PD-VH vs. PD-nonVH). Dunn’s multiple comparison test was used for post-hoc analyses.

We calculated Pearson’s coefficient for the correlations between the visual detection threshold on one hand and the neurological and neuropsychological scores on the other.

A *P*-value below 0.05 (after Bonferroni correction) was considered to be statistically significant.

### fMRI analyses

fMRI data acquisition and preprocessing were summarized in Supplementary file 2.

### Whole-brain ANCOVA

To directly compare the groups in terms of the activation associated with visual detection at the threshold [ST-UT], a whole-brain two-factor analysis of covariance (ANCOVA) was performed using condition estimates (beta values) from a first-level random effect (RFX) GLM analysis of all the participants (n = 51), with fMRI condition (ST, UT, SNT, UNT, OT) as the within-group factor and group as the between-group factor (HC, PD-VH, PD-nonVH)]. Demographic and clinical variables for which between-group differences were observed (HDRS, TMTB/TMTA and the Stroop error score), were used as additional covariates.

The post-hoc contrast analyses compared brain activation during conscious access to visual inputs ([ST-UT]) in pairs of groups. The first post-hoc analysis explored disease-specific brain reorganization by comparing HCs with all PD patients (i.e. the pooled PD-VH and PD-nonVH subgroups). The second analysis was intended to highlight differences associated with susceptibility to hallucinations in PD, and thus compared the PD-VH and PD-nonVH subgroups.

A Pearson correlation was performed between the beta-value of the involved areas and the severity, frequency and intensity of visual hallucinations (visual analog scales and UM-PDHQ visual modality). A *p*-value< 0.05 (after Bonferroni correction) was considered as statistically significant.

### Whole-brain random-effects general linear models

In order to detail the activation pattern in each group, 3 whole-brain RFX-GLMs were built (one for each group). Results are presented in Supplementary file 3.

Statistical analyses were corrected at the cluster level by the use of the “ClusterThresh” plugin in BrainVoyager. This method is based on the assumption that areas of activity have a tendency to stimulate signal changes over spatially contiguous groups of voxels rather than over isolated voxels.

The computation of the minimum cluster threshold is accomplished via MonteCarlo simulation of the random process of image generation, followed by the injection of spatial correlations between neighbouring voxels and voxel intensity thresholding. Starting from a manually adjusted voxel-level probability threshold, set here at *P* < 0.01, a minimum cluster size threshold is automatically set which yields 5% protection against false positive detection at the cluster level (confidence level of alpha = 0.05). By applying the cluster size threshold, the resulting map becomes “corrected for multiple comparisons” at a desired confidence level (set here at alpha = 0.05).

## Additional Information

**How to cite this article**: Lefebvre, S. *et al*. Hallucinations and conscious access to visual inputs in Parkinson’s disease. *Sci. Rep.* **6**, 36284; doi: 10.1038/srep36284 (2016).

**Publisher’s note:** Springer Nature remains neutral with regard to jurisdictional claims in published maps and institutional affiliations.

## Supplementary Material

Supplementary Information

## Figures and Tables

**Table 1:** Demographic and clinical characteristics of the three subject groups

	PD without VHs (n = 16) Mean *(SD)*	PD with minor VHs (n = 18) Mean *(SD)*	Healthy controls (n = 17) Mean *(SD)*	p value	Post-hoc test
Demographics					
Gender (female/male)	4/12	7/11	7/10		
Age (y)	62.69 *(4.09)*	63.50 *(5.94)*	62.76 *(4.19)*	0.86	NA
Educational level (y of full-time education)	13.38 *(4.19)*	12.44 *(3.33)*	13.76 *(1.92)*	0.47	NA
Clinical characteristics					
Disease duration (y)	8.00 *(5.74)*	9.06 *(4.11)*	NA	0.54	NA
MDS-UPDRS3 score	21.81 *(7.93)*	25 *(8.44)*	NA	0.27	NA
Hoehn & Yahr stage (Median; first-third quartile)	*2;2–2*	*2;2–2*	NA	0.09	NA
Side of onset	9 R/5 L+2 undefined	10 R/6 L + 2 undefined	NA		
UM-PDHQ visual modality	NA	5.78 *(2.07)*	NA		
VH frequency (0 to 20 on a visual analog scale)	NA	3.05 *(1.99)*	NA		
VH severity (0 to 20 on a visual analogue scale)	NA	1.56 *(2.17)*	NA		
Medication					
LEDD (mg/day)	804.25 *(297.39)*	859.72 *(411.08)*	NA	0.66	NA
Agonist-LEDD (mg/day)	184.71 *(182.49)*	166.83 *(117.78)*	NA	0.58	NA
Neuropsychiatric					
Hamilton Depression Rating Scale	3.06 *(2.88)*	3.56 *(2.99)*	1.12 *(1.45)*	0.02	2≠3
Score on the PAS: persistent anxiety	2.81 *(3.60)*	5.28 *(5.57)*	1.35 *(2.06)*	0.02	2≠3
Score on the PAS: episodic anxiety	0.31 *(0.60)*	0.83 *(1.58)*	0.00 *(0.00)*	0.05	
Score on the PAS: avoidance	1.00 *(1.55)*	1.00 *(1.64)*	0.06 *(0.24)*	0.06	NA
Lille Apathy Rating Scale	−27.13 *(4.95)*	−26.56 *(3.48)*	−28.35 *(4.47)*	0.46	NA
Visual detection threshold (ms)	163.38 *129.72*	175.17 *124.12*	69.29 *55.99*	0.01	1≠3; 2≠3
Overall efficiency					
Mini Mental State Examination (out of 30)	28.88 *1.20*	28.00 *1.24*	28.47 *1.70*	0.20	NA
Mattis Dementia Rating Scale (out of 144)	140.44 *3.22*	140.33 *2.38*	142.06 *1.71*	0.09	NA
Attention/Working memory					
WAIS-R forward digit span	6.06 *0.99*	5.67 *0.91*	5.47 *1.07*	0.22	NA
WAIS-R backward digit span	4.31 *0.70*	4.22 *0.94*	4.35 *0.93*	0.90	NA
SDMT: number in 90 sec	48.38 *9.74*	41.39 *12.05*	47.06 *5.39*	0.08	NA
Episodic Memory					
HVLT Learn1 (out of 12)	6.50 *1.93*	6.11 *2.32*	7.18 *1.91*	0.32	NA
HVLT Learn total (out of 36)	26.63 *4.18*	25.17 *5.08*	28.82 *3.92*	0.06	NA
HVLT long-term retention (%)	92.20 *12.27*	95.91 *11.26*	96.84 *6.24*	0.37	NA
HVLT recognition hits (out of 12)	11.69 *0.48*	11.50 *0.79*	11.76 *0.44*	0.41	NA
HVLT number of intrusions	1.81 *1.28*	2.22 *2.84*	1.59 *2.69*	0.73	NA
Executive functions					
Trail Making Test (time B/time A)	2.48 *0.64*	3.58 *1.14*	3.85 *1.44*	<0.01	1 ≠ 2; 1 ≠ 3
Stroop: interference index	1.64 *0.40*	1.79 *0.30*	1.78 *0.27*	0.31	NA
Stroop: errors	0.87 *0.96*	2.78 *3.17*	1.00 *1.50*	0.02	1≠2
Phonemic fluency: naming in 60 sec.	14.75 *3.57*	16.61 *5.24*	16.71 *3.79*	0.35	NA
Alternating fluency: naming in 60 sec	13.31 *3.07*	13.11 *3.50*	13.94 *3.29*	0.74	NA
language					
Boston naming test (out of 15)	12.94 *1.57*	12.44 *1.46*	13.35 *1.54*	0.22	NA
Animal naming in 60 sec	20.88 *4.69*	20.22 *3.77*	20.71 *3.27*	0.88	NA
Visuospatial functions					
Judgment of line orientation (out of 15)	13.31 *2.24*	12.28 *1.45*	13.41 *1.12*	0.09	NA
Visuoconstruction (out of 12)	11.06 *1.12*	11.33 *1.08*	10.71 *0.69*	0.18	NA

VH: visual hallucination; MDS UPDRS3: Movement Disorders Society sponsored revision of the Unified Parkinson’s Disease Rating Scale-Part III (severity of motor symptoms); UM-PDHQ: University of Miami Parkinson’s Disease Hallucination Questionnaire; LEDD: levodopa equivalent daily dose; PAS: Parkinson Anxiety Scale; WAIS-R: Wechsler Adult Intelligence Scale-Revised; SDMT: symbol digit modalities test; HVLT: Hopkins Verbal Learning Test; NA: not applicable.

**Table 2:** Whole brain ANCOVA.

	Brain area	BA	mean x	mean y	mean z	mm^3^	Activation peak
							t value
[ST-UT] HCs vs PD patients: t(200) = 2.59, *P* = 0.01, corrected at the cluster level							
PD patients > HCs	R thalamus		22	−16	12	3 819	−4.6
	R precuneus	7	22	−53	49	1 515	−3.17
HCs > PD patients	L PMd	6	−20	−10	51	1 676	3,26
[ST-UT] PD-VH vs PD-nonVH: t(200) = 2.59, *P* = 0.01, corrected at the cluster level							
PD-VH > PD-nonVH	R cerebellum		28	−57	−39	259	3,30
	R occipital cortex	18	21	−72	−1	139	3,27
	R prefrontal cortex	9	9	30	52	140	2.77
PD-nonVH > PD-VH	L cingulate cortex	31	−20	−26	35	6 433	−4,17
	L caudate nucleus	−23	−27	24	828	−3,5	
	L temporal cortex	22	−47	−42	4	154	−3,15
	L occipital cortex	19	−35	−69	−8	178	−3,13

PD-nonVH: PD patients without visual hallucinations; PD-VH: PD patients with visual hallucinations; HC: healthy controls; ST: seen at the threshold; UT: unseen at the threshold; SNT: seen not at the threshold; UNT: unseen not at the threshold; DLPFC: dorsolateral prefrontal cortex; PMd: dorsal premotor cortex; BA: Brodmann area; R: right; L: left.
