# Supplementary material for: Hallucinations and conscious access to visual inputs in Parkinson’s disease
Source: Sci Rep. 2016 Nov 14;6:36284. doi: 10.1038/srep36284 (PMC5107911; doi:10.1038/srep36284)
Supplement: Supplementary Information [file srep36284-s1.pdf]

Supplemental informations

Hallucinations and conscious access to visual inputs in Parkinson's disease

Stéphanie Lefebvre, PhD^[1,2], Guillaume Baille, MD^[4], Renaud Jardri MD, PhD[1,2] Lucie Plomhause, PhD[3], Sébastien Szaffarczyk[1,2], Luc Defebvre, MD, PhD [3,4], Pierre Thomas MD, PhD [1,2], Christine Delmaire, MD, PhD [3,5], Delphine Pins, PhD[1,2], Kathy Dujardin*, PhD [3,4]

Supplementary file 1*: **Patients' symptoms evaluation***

The presence of VHs was confirmed by the hallucination item of the Neuropsychiatric Inventory-Clinician [1]. The severity and frequency of VHs were rated on a visual analog scale, and the intensity of VHs was assessed by using the University of Miami Parkinson's Disease Hallucinations Questionnaire [2]

Doses of antiparkinsonian medication were converted to the levodopa equivalent daily dose by applying the algorithm developed by Tomlinson et al. [3]. The severity of motor symptoms was assessed on the Movement Disorders Society - Unified Parkinson's Disease Rating Scale [4](MDS-UPDRS) (part III), and the disease stage was assessed with the Hoehn & Yahr score [5]. The severity of depression, apathy and anxiety symptoms was quantified with the 17-item Hamilton Depression Rating Scale [6], the Lille Apathy Rating Scale [7] and the Parkinson Anxiety Scale [8], respectively. Hand dominance was determined according to the Edinburgh Handedness Inventory [9]. Furthermore, a battery of neuropsychological tests was administrated to the participants

1 de Medeiros, K. *et al.* The Neuropsychiatric Inventory-Clinician rating scale (NPI-C): reliability and validity of a revised assessment of neuropsychiatric symptoms in dementia. *Int Psychogeriatr* **22**, 984-994, doi:10.1017/S1041610210000876 S1041610210000876 [pii] (2010).

2 Papapetropoulos, S. *et al.* A questionnaire-based (UM-PDHQ) study of hallucinations in Parkinson's disease. *BMC Neurol* **8**, 21, doi:10.1186/1471-2377-8-21
1471-2377-8-21 [pii] (2008).
3 Tomlinson, C. L. *et al.* Systematic review of levodopa dose equivalency reporting in Parkinson's disease. *Mov Disord* **25**, 2649-2653, doi:10.1002/mds.23429 (2010).
4 Goetz, C. G. *et al.* Movement Disorder Society-sponsored revision of the Unified Parkinson's Disease Rating Scale (MDS-UPDRS): scale presentation and clinimetric testing results. *Mov Disord* **23**, 2129-2170, doi:10.1002/mds.22340 (2008).
5 Hoehn, M. M. & Yahr, M. D. Parkinsonism: onset, progression, and mortality. 1967. *Neurology* **57**, S11-26 (2001).
6 Hamilton, M. A rating scale for depression. *J Neurol Neurosurg Psychiatry* **23**, 56-62 (1960).
7 Sockeel, P. *et al.* The Lille apathy rating scale (LARS), a new instrument for detecting and quantifying apathy: validation in Parkinson's disease. *J Neurol Neurosurg Psychiatry* **77**, 579-584, doi:77/5/579 [pii]
10.1136/jnnp.2005.075929 (2006).
8 Leentjens, A. F. *et al.* The Parkinson Anxiety Scale (PAS): development and validation of a new anxiety scale. *Mov Disord* **29**, 1035-1043, doi:10.1002/mds.25919 (2014).
9 Oldfield, R. C. The assessment and analysis of handedness: the Edinburgh inventory. *Neuropsychologia* **9**, 97-113 (1971).

Supplementary file <u>**2**</u>:***fMRI data acquisition and preprocessing***

Acquisition

A 3T MRI scanner (Intera Achieva, Philips Medical Systems, Philips Healthcare P.O., Best, The Netherlands) with a 12-channel head coil was used to acquire the three-dimensional (3D) T1 anatomical data and fMRI data. The fMRI data were acquired using a “principles of echo with train of observations” (PRESTO) sequence with the following parameters: repetition time = 1000ms, interslice time = 22ms, flip angle = 9, matrix size = 64 x 64, field of view = 206 x 206 x 153 mm, slice thickness = 3.4 mm, number of slices = 45, number of volumes = 1215, duration = 20 min. A 3D T1-weighted dataset encompassing the whole brain was used to obtain detailed anatomic data (voxels: 1 mm^3), using the following parameters: repetition time = 7ms; flip angle = 9; matrix size = 64 x 64; field of view = 256 x 256 x 160 mm; slice thickness = 1 mm; number of slices = 176.

Preprocessing

MRI data were analyzed using BrainVoyager QX software (Version 2.8, Brain Innovation, Maastricht, The Netherlands). The pre-processing of the functional data consisted of time-domain high-pass filtering (i.e. the removal of frequencies below three cycles/run) and 3D motion correction for head movements using a rigid body algorithm. Coregistrations between functional runs and 3D-T1 weighted scans of each patient were performed automatically using gradient-driven affine transformations with 9 alignment parameters (3 translations, 3 rotations and 3 scales), and then manually corrected (if needed). To allow group analyses, all anatomical and functional volumes were spatially normalized in Talairach space [1]. fMRI scans were smoothed in the spatial domain with a 6mm Gaussian filter.

Functional data were analyzed using a general linear model (GLM) with predictors based on specific experimental conditions (ST, UT, SNT, UNT and OT). Beta weights were

used to measure the predictors' potential contributions to each voxel time course.

1 Talairach J, T. P. *Co-planar stereotaxic atlas of the human brain. 3-dimensional proportional system: an approach to cerebral imaging.* . (Stuttgart: Thieme Verlag, 1988).

<u>Supplementary file 4:</u>* *Whole-brain RFX GLM analyses

For each model, the brain activation associated with each condition was explored at the whole-brain level by using the following contrasts: (i) [ST+SNT], which refers to all the “seen” trials and reflects conscious vision; (ii) [UT+UNT], which refers to all the “unseen” trials and reflects unconscious vision; and (iii) [ST-UT], which probes brain activation associated with conscious access to visual inputs.

The whole-brain activation patterns detected by the three RFX analyses (one for each group) are shown in Figure A and Table A. Visual inspection of the Figure shows that the activation patterns for the “seen” [ST+SNT] and “unseen” [UT+UNT] conditions were similar in each of the three groups. In all 3 groups, the “seen” and “unseen” trials were associated with marked activation of the left motor cortex, bilateral premotor cortices, right insula, and right (pre)frontal, temporal and inferior parietal cortices. In contrast, the right precuneus cortex and left occipital cortex were not involved in PD-VH patients. The PD patients as a whole differed from the HCs in several specific respects; the patients activated the left insula and displayed less deactivation in other areas.

Furthermore, differences in the activation related to conscious access to visual inputs [ST-UT] were observed in each of the three groups. This suggested that the presentation of a stimulus (whether seen or not) induced similar processing steps, whereas conscious access to visual inputs involved the specific activation of a neural network.

<u>Region of interest analyses</u>

To directly probe susceptibility to hallucinations in Parkinson’s disease, we directly compared the PD-VH and PD-nonVH groups in terms of regions of interest (ROIs). To avoid non-independent correlations [1], we used the functional ROI activated in HCs during [ST-UT]

(p=0.01, corrected at the cluster level), to compare the blood-oxygen-level-dependent activation in the two patient groups (PD-VH vs. PD-nonVH). This analysis revealed a statistically significant difference for the right prefrontal cortex (BA 8) only (beta weights for PD-nonVH: -27.675; PD-VH: 28.88; t(32) = 2.06, *P* = 0.048), suggesting hyperactivation of this area during the [ST-UT] condition in participants in the PD-VH group.

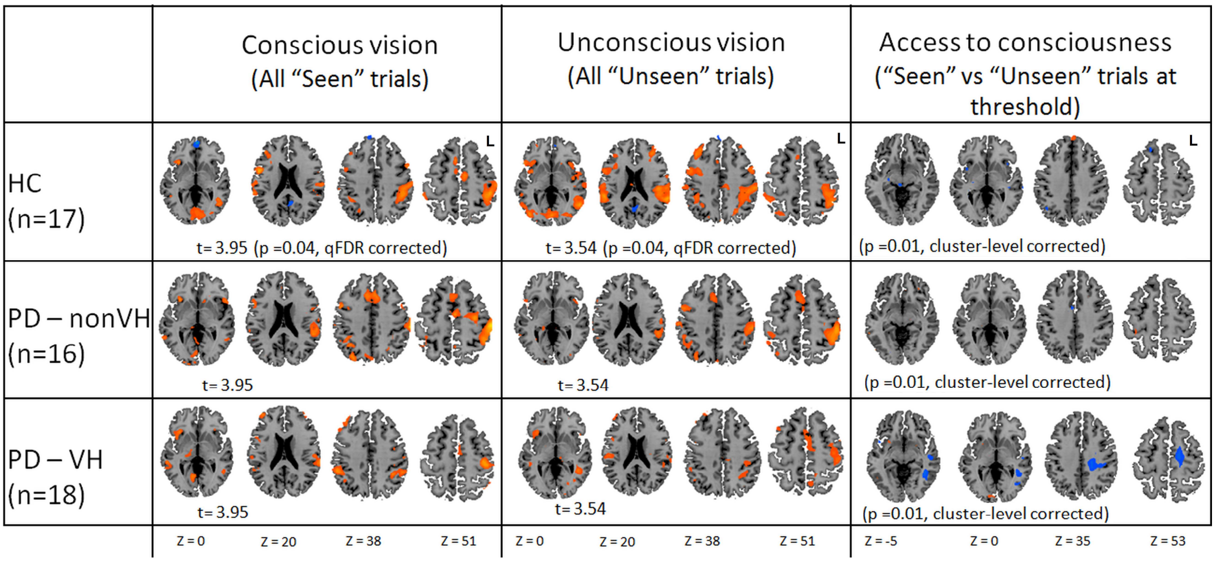

<u>Figure A</u> : Activation patterns in each of the 3 groups

Whole-brain activation in each of the 3 groups and the 3 main conditions: conscious vision (t=3.95), unconscious vision (t=3.54) and conscious access to visual inputs (p =0.01, corrected at the cluster level).

PD-nonVH: Parkinson’s disease patients without visual hallucinations; PD-VH: Parkinson’s disease patients with visual hallucinations; HC: healthy controls; L: left.

***<u>Table A:</u>* Whole-brain RFX GLM analysis**

		Brain area	BA	mean x	mean y	mean z	mm³	activation peak t value
[ST+ SNT] t=3.95								
HCs	Activation	*R inferior frontal cortex*	*44*	*50*	*5*	*19*	*2 999*	*8.24*
		R PMv	*6*	*46*	*3*	*39*	*249*	*4.57*
		R precuneus	*7*	*26*	*-64*	*34*	*422*	*5.79*
		R SMA	*6*	*6*	*3*	*51*	*1 067*	*6.16*
		R DLPFC	*9*	*36*	*37*	*26*	*1 814*	*6.67*
		R insula	*13*	*32*	*21*	*7*	*2232*	*5.94*
		R superior temporal cortex	*22*	*56*	*-29*	*20*	*2 306*	*5.65*
		R inferior parietal cortex	*40*	*44*	*-42*	*44*	*678*	*5.32*
		R occipital cortex	*17-18-19*	*19*	*-77*	*1*	*6 548*	*6.53*
		R brainstem midbrain		*4*	*-17*	*-13*	*354*	*5.04*
		R Cerebellum		*29*	*-58*	*-22*	*3 441*	*5.72*
		L SMA	*6*	*-6*	*-11*	*52*	*858*	*5.61*
		L precuneus	*7*	*-25*	*-64*	*32*	*363*	*6.70*
		L temporal cortex	*41*	*-47*	*5*	*8*	*264*	*4.54*
		L PMv	*6*	*-55*	*5*	*32*	*1 400*	*7.04*
		L M1	*4*	*-42*	*-29*	*48*	*4 639*	*6.49*
		L PMd	*6*	*-39*	*-14*	*59*	*463*	*4.32*
		L somatosensory cortex	*3*	*-37*	*-40*	*51*	*1 164*	*6.26*
		L inferior parietal cortex	*40*	*-42*	*-40*	*36*	*3 767*	*6.34*
		L occipital cortex	*17-18-19*	*-39*	*-64*	*-21*	*8 901*	*5.64*
		L thalamus		*-15*	*-2*	*11*	*153*	*4.49*
		L cerebellum		*-29*	*-62*	*-23*	*9 764*	*7.07*
	Deactivation	Bilateral prefrontal cortex	10	0	55	3	1 197	6.52
		Bilateral posterior cingulate cortex	31	-3	-56	15	1 407	6.19
		R prefrontal cortex	8	20	36	48	166	4.58
		L prefrontal cortex	8	-9	49	44	97	4.58
PD-nonVH	Activation	*Bilateral SMA*	*6*	*0*	*12*	*46*	*4 524*	*6.18*
		R middle temporal cortex	*21*	*59*	*-50*	*0*	*201*	*4.71*
		R DLPFC	*9*	*48*	*11*	*23*	*1 976*	*6.57*
		R parietal cortex	*5*	*38*	*-42*	*58*	*135*	*4.49*

		R insula	*13*	*33*	*20*	*3*	*434*	*6.33*
		R precuneus	*19*	*27*	*-75*	*40*	*1 139*	*5.92*
		R precuneus	*7*	*19*	*-62*	*30*	*230*	*5.62*
		R PMd	*6*	*19*	*-4*	*67*	*95*	*5.47*
		R thalamus		*15*	*-22*	*13*	*438*	*5.82*
		R occipital		*5*	*-77*	*2*	*288*	*4.67*
		R brainstem pons		*5*	*-28*	*-28*	*138*	*4.69*
		R inferior parietal cortex	*40*	*38*	*-47*	*42*	*459*	*5.53*
		L inferior parietal cortex	*40*	*-43*	*-38*	*49*	*2 026*	*6.91*
		L M1	*4*	*-49*	*-25*	*48*	*2 666*	*8.05*
		L PMd	*6*	*-27*	*-13*	*55*	*1 466*	*6.09*
		L middle temporal cortex	*21*	*-52*	*-30*	*18*	*2 353*	*6.64*
		L insula	*13*	*-34*	*19*	*8*	*1 050*	*6.55*
		L occipital cortex		*-45*	*-58*	*-12*	*372*	*7.89*
		L cerebellum		*-22*	*-63*	*-21*	*2 787*	*6.45*
	Deactivation	N/A						
PD-VH	Activation	*R temporal cortex*	*22*	*63*	*-40*	*14*	*91*	*4.75*
		R temporal cortex	*21*	*59*	*-21*	*15*	*506*	*4.82*
		R inferior parietal cortex	*40*	*50*	*-42*	*27*	*5 834*	*6.47*
		R inferior frontal cortex	*44*	*49*	*13*	*16*	*707*	*5.24*
		R insula	*13*	*36*	*17*	*4*	*1 976*	*6.29*
		R SMA	*6*	*5*	*-1*	*59*	*58*	*4.65*
		R thalamus		*11*	*-15*	*5*	*3 202*	*6.08*
		R DLPFC	*9*	*38*	*42*	*28*	*3 186*	*5.74*
		R occipital cortex	*17-18-19*	*10*	*-71*	*-2*	*2 136*	*5.74*
		R cerebellum		*35*	*-59*	*-25*	*321*	*5.13*
		L SMA	*6*	*-4*	*-3*	*50*	*453*	*4.92*
		L insula	*13*	*-31*	*23*	*6*	*625*	*5.11*
		L inferior parietal cortex	*40*	*-45*	*-48*	*40*	*2 614*	*5.89*
		L DLPFC	*9*	*-39*	*30*	*34*	*74*	*4.59*
		L PMv	*6*	*-39*	*-6*	*38*	*60*	*4.75*
		L temporal cortex	*42*	*-55*	*-30*	*16*	*5 831*	*6*
		L M1	*4*	*-38*	*-24*	*54*	*1 602*	*6.47*
		L PMd	*6*	*-38*	*-15*	*58*	*984*	*6.14*
		L somatosensory cortex	*3*	*-42*	*-29*	*48*	*756*	*6.17*

		L prefrontal cortex	*10*	*-36*	*48*	*23*	*310*	*4.98*
		L cerebellum		*-22*	*-67*	*-26*	*959*	*5.13*
	Deactivation	Bilateral prefrontal cortex	8	-1	46	42	81	4.41
[UT+ UNT] t=3.54								
HCs	Activation	*R DLPFC*	*9*	*34*	*34*	*33*	*3 528*	*6.26*
		R SMA	*6*	*5*	*13*	*48*	*553*	*4.45*
		R temporal cortex	*13*	*56*	*-39*	*19*	*3 211*	*5.63*
		R insula	*13*	*35*	*19*	*11*	*3 380*	*6.02*
		R PMv	*6*	*48*	*5*	*31*	*4 210*	*6.95*
		R parietal superior cortex	*7*	*40*	*-42*	*45*	*3 479*	*5.13*
		R occipital cortex	*17-18-19*	*19*	*-77*	*1*	*6 548*	*5.53*
		R thalamus		*16*	*-12*	*14*	*446*	*5.76*
		R cerebellum		*19*	*-60*	*-19*	*10 017*	*6.75*
		L M1	*4*	*-42*	*-29*	*48*	*5 039*	*7.49*
		L somatosensory cortex	*3*	*-37*	*-40*	*51*	*2 164*	*5.26*
		L inferior parietal cortex	*40*	*-42*	*-40*	*36*	*1 767*	*5.34*
		L DLPFC	*9*	*-29*	*32*	*14*	*5 496*	*7.11*
		L precuneus	*7*	*-25*	*-63*	*35*	*442*	*5.32*
		L hippocampal complex		*-39*	*-24*	*-9*	*146*	*4.56*
		L PMv	*6*	*-51*	*4*	*34*	*1 114*	*4.72*
		L occipital cortex	*17-18-19*	*-39*	*-64*	*-21*	*9 962*	*6.64*
		L temporal cortex	*21*	*-63*	*-28*	*10*	*11 374*	*7.18*
		L PMd	*6*	*-39*	*-14*	*59*	*563*	*4.99*
		L caudate nucleus		*-19*	*19*	*4*	*102*	*4.55*
		L cerebellum		*-27*	*-63*	*-24*	*11 826*	*7.33*
	Deactivation	Bilateral posterior cingulate cortex	31	0	-56	13	880	5.51
		L temporal cortex	39	-49	-70	25	75	4.47
PD-nonVH	Activation	*Bilateral SMA*	*6*	*2*	*11*	*50*	*4 673*	*4.60*
		R temporal cortex	*21*	*58*	*-45*	*8*	*1 677*	*4.68*
		R PMv	*6*	*46*	*3*	*37*	*1 543*	*5.02*
		R insula	*13*	*44*	*10*	*6*	*2 546*	*5.20*
		R PMd	*6*	*26*	*-22*	*68*	*130*	*4.54*
		R occipital cortex	*18*	*22*	*-92*	*5*	*219*	*4.54*
		R hippocampal complex		*17*	*-34*	*0*	*167*	*4.34*
		R inferior parietal cortex	*40*	*45*	*-46*	*39*	*5 200*	*5.91*

		R caudate nucleus		*7*	*7*	*11*	*148*	*4.08*
		R cerebellum		*26*	*-46*	*-20*	*429*	*4.28*
		L precuneus	*7*	*-3*	*-78*	*39*	*76*	*4.18*
		L insula	*13*	*-38*	*15*	*2*	*409*	*4.28*
		L PMv	*6*	*-53*	*10*	*29*	*305*	*4.09*
		L temporal cortex	*21*	*-54*	*-26*	*-3*	*77*	*4.28*
		L M1	*4*	*-39*	*-31*	*53*	*4 179*	*6.24*
		L PMd	*6*	*-29*	*-18*	*62*	*6 421*	*6.62*
		L inferior parietal cortex	*40*	*-48*	*-33*	*43*	*1 793*	*6.24*
		L caudate nucleus		*-5*	*4*	*10*	*137*	*3.99*
		L cerebellum		*-18*	*-70*	*-24*	*2 575*	*4.91*
	Deactivation	N/A						
PD-VH	Activation	*Bilateral SMA*	*6*	*-2*	*0*	*50*	*2 985*	*4.86*
		R temporal cortex	*22*	*52*	*-35*	*2*	*1 794*	*5.35*
		R temporal cortex	*21*	*-22*	*15*	*5*	*2 342*	*4.65*
		R PMv	*6*	*40*	*11*	*48*	*556*	*4.37*
		R DLPFC	*9*	*35*	*48*	*26*	*1 638*	*5.12*
		R insula	*13*	*36*	*20*	*5*	*2 007*	*5.27*
		R inferior parietal cortex	*40*	*49*	*-44*	*32*	*2 223*	*5.40*
		R thalamus		*-13*	*-21*	*11*	*462*	*4.46*
		R cerebellum		*10*	*-66*	*-14*	*586*	*4.50*
		L cingular cortex	*31*	*-11*	*-29*	*38*	*1 102*	*4.79*
		L PMd	*6*	*-35*	*-16*	*51*	*3 553*	*5.35*
		L M1	*4*	*-39*	*-25*	*49*	*3 858*	*4.88*
		L precuneus	*7*	*-11*	*-55*	*48*	*573*	*4.33*
		L inferior parietal cortex	*40*	*-30*	*-50*	*35*	*843*	*4.79*
		L insula	*13*	*-29*	*24*	*6*	*243*	*4.24*
		L inferior frontal cortex	*44*	*-49*	*6*	*14*	*710*	*4.71*
		L temporal cortex	*41*	*-55*	*-22*	*13*	*2 535*	*4.97*
		L occipital cortex	*17*	*-18*	*-75*	*10*	*311*	*4.16*
		L brainstem red nucleus		*-9*	*-23*	*-3*	*192*	*4.78*
		L thalamus		*-13*	*-21*	*11*	*462*	*4.46*
		L cerebellum		*-23*	*-64*	*-21*	*6 773*	*5.09*
	Deactivation	N/A						
[ST- UT] p=0.01, corrected at the cluster level								

HCs	ST > UT	*Bilateral DLPFC*	*9*	*-5*	*55*	*34*	*277*	*3.80*
	UT > ST	R DLPFC	9	50	10	29	56	3.39
		R parietal cortex	39	39	-60	33	90	3.26
		R insula	13	38	-6	19	45	3.34
		R claustrum		35	5	1	28	3.23
		R putamen		27	-21	9	134	3.31
		R hippocampal complex		23	-19	-10	129	4.03
		R prefrontal cortex	8	13	33	45	26	2.73
		R superior colliculus		2	-28	-7	118	3.80
		L temporal cortex	21	-67	-29	2	36	3.64
PD-nonVH	ST> UT	*R M1*	*4*	*33*	*-35*	60	173	3.58
		R cerebellum		*30*	*-76*	-23	30	4.78
		L brainstem substantia nigra		*-15*	*-19*	-8	33	4.16
		L prefrontal	*8*	*-21*	*21*	37	25	3.37
	UT > ST	L M1	4	-36	-34	55	25	3.80
PD-VH	ST > UT	*R frontal cortex*	*46*	*52*	*27*	*25*	*113*	*3.65*
		R precuneus	*31*	*24*	*-74*	*16*	*1 458*	*3.83*
		R cerebellum		*46*	*-63*	*-27*	*76*	*3.47*
	UT > ST	L inferior parietal cortex	40	-40	-31	40	493	3.66
		L temporal cortex	38	-45	11	-10	129	3.81
		L SMA	6	-17	-12	49	1 226	3.49
		L cingular cortex	31	-18	-29	37	2 003	3.70
		L prefrontal	9	52	19	28	115	3.71
		L hippocampal complex		-40	-47	-3	697	3.75

PD-nonVH: Parkinson's disease patients without visual hallucinations; PD-VH: Parkinson's disease patients with visual hallucinations; HC: Healthy controls; ST: seen at the threshold; UT: unseen at the threshold; SNT: seen not at the threshold; UNT: unseen not at the threshold; M1: primary motor cortex; SMA: supplementary motor area; DLPFC: dorsolateral prefrontal cortex; PMd: dorsal premotor cortex; PMv: ventral premotor cortex; BA: Brodmann area.

1 Vul, E., Harris, C., Winkielman, P. & Pashler, H. Puzzlingly High Correlations in fMRI Studies of Emotion, Personality, and Social Cognition. *Perspect Psychol Sci* **4**, 274-290, doi:10.1111/j.1745-6924.2009.01125.x
4/3/274 [pii] (2009).
